# Only Slight Impact of Predicted Replicative Capacity for Therapy Response Prediction

**DOI:** 10.1371/journal.pone.0009044

**Published:** 2010-02-03

**Authors:** Hendrik Weisser, André Altmann, Saleta Sierra, Francesca Incardona, Daniel Struck, Anders Sönnerborg, Rolf Kaiser, Maurizio Zazzi, Monika Tschochner, Hauke Walter, Thomas Lengauer

**Affiliations:** 1 Computational Biology and Applied Algorithmics, Max Planck Institute for Informatics, Saarbrücken, Germany; 2 Institute of Virology, University of Cologne, Cologne, Germany; 3 Informa s.r.l., Rome, Italy; 4 Retrovirology Laboratory, CRP-Santé, Strassen, Luxembourg; 5 Department of Medicine, Division of Infectious Diseases, Karolinska Institute, Stockholm, Sweden; 6 Department of Molecular Biology, University of Siena, Siena, Italy; 7 Institute of Clinical and Molecular Virology, University of Erlangen, Erlangen, Germany; Institut Pasteur, France

## Abstract

**Background:**

Replication capacity (RC) of specific HIV isolates is occasionally blamed for unexpected treatment responses. However, the role of viral RC in response to antiretroviral therapy is not yet fully understood.

**Materials and Methods:**

We developed a method for predicting RC from genotype using support vector machines (SVMs) trained on about 300 genotype-RC pairs. Next, we studied the impact of predicted viral RC (pRC) on the change of viral load (VL) and CD4^+^ T-cell count (CD4) during the course of therapy on about 3,000 treatment change episodes (TCEs) extracted from the EuResist integrated database. Specifically, linear regression models using either treatment activity scores (TAS), the drug combination, or pRC or any combination of these covariates were trained to predict change in VL and CD4, respectively.

**Results:**

The SVM models achieved a Spearman correlation (ρ) of 0.54 between measured RC and pRC. The prediction of change in VL (CD4) was best at 180 (360) days, reaching a correlation of *ρ* = 0.45 (*ρ* = 0.27). In general, pRC was inversely correlated to drug resistance at treatment start (on average *ρ* = −0.38). Inclusion of pRC in the linear regression models significantly improved prediction of virological response to treatment based either on the drug combination or on the TAS (t-test; p-values range from 0.0247 to 4 10^−6^) but not for the model using both TAS and drug combination. For predicting the change in CD4 the improvement derived from inclusion of pRC was not significant.

**Conclusion:**

Viral RC could be predicted from genotype with moderate accuracy and could slightly improve prediction of virological treatment response. However, the observed improvement could simply be a consequence of the significant correlation between pRC and drug resistance.

## Introduction

Modern therapy for patients infected with the human immunodeficiency virus type 1 (HIV-1) aims to reduce viral replication by blocking a number of targets. Viral entry can be blocked by either a fusion inhibitor binding to the viral gp41 protein or by a coreceptor antagonist that binds to the CCR5 coreceptor and therefore prevents the CCR5-using viruses from entering the host cell. Reverse transcriptase (RT) inhibitors are subdivided into two classes depending on their mechanism of action. Nucleoside reverse transcriptase inhibitors (NRTIs) are nucleoside analogs that block chain elongation after their incorporation into newly synthesized DNA. Non-nucleoside reverse transcriptase inhibitors (NNRTIs) bind to the viral RT and allosterically hinder DNA polymerization by impairing the mobility of particular RT domains [1]. Integrase inhibitors impede the transfer of the newly synthesized viral DNA into the host genome. Protease (PR) inhibitors (PIs) prevent the maturation of infectious particles by blocking cleavage of gag-pol polyproteins by the viral protease [2].

The extraordinary capability of HIV to escape from drug pressure by developing resistance mutations promotes the continuous development of new antiretroviral drugs. HIV RT lacks a proof-reading mechanism, resulting in the high mutation rate of HIV (3.4 • 10^−5^ mutations per base per cycle of replication [3]). The high error rate coupled with a short generation time leads to a rapid selection of resistance mutations reducing susceptibility to drugs, sometimes within days [4]. To maximally suppress viral replication and minimize the rapid emergence of drug resistance mutations, modern anti-HIV therapy combines several compounds from different drug classes for achieving different selective pressures and higher antiviral potency. This practice, called highly active antiretroviral therapy (HAART), achieves a dramatic delay in disease progression to AIDS and death [5].

However, after the introduction of HAART, the prevalence of drug resistance in therapy-experienced – but also in therapy-naïve – patients increased and raised the need for assessing the viral susceptibility to each compound before administration [6], [7]. Drug resistance can be determined functionally by recombinant virus assays testing viral replication against every compound *in vitro* [8]–[10]. However, obtaining the drug resistance phenotype is labour- and cost-intensive. Thus, sequencing of the relevant regions of the HIV genome for detecting drug resistance associated mutations has become standard-of-care. In contrast to phenotypic information, data resulting from genotyping is complex and hard to interpret. Several tools have been developed for interpretation of the genotype. Rules-based algorithms apply sets of rules, which were handcrafted by experts to assess resistance against a single compound [11], [12]. Approaches based on bioinformatics methods try to predict the fold-change in resistance from genotype [13], [14]. Recent bioinformatics approaches go even further and predict *in vivo* response to antiretroviral combination therapy [15], [16].

Resistance mutations bear a clear advantage for the virus in the presence of drugs. They alter the viral proteins in a way that they remain functional even in the presence of the given drugs. However, most of the resistance mutations lead to disadvantages for the virus in the absence of drugs, as they are neither observed in their natural, drug-free environment, nor usually after interruption of therapy [17]. In other words, being able to replicate in the presence of drugs comes at the cost of reduced function of the target protein with respect to the wild type protein. Therefore, resistance has an impact on viral fitness, which stands more generally for the ability to yield progeny virus in a given environment, as compared to a reference strain in the same environment [18]. A number of resistance mutations are indeed termed compensatory or secondary drug resistance mutations since they restore at least partially the enzyme function possibly compromised by the true resistance mutation(s). The efficacy of the viral replication machinery in drug resistant strains thus depends on the interaction between impairing and compensating effects and influences the velocity of viral replication – captured by the term replicative capacity (RC). RC determination has been faced in numerous ways and can be assessed by counting the number of newly assembled infectious viruses within a fixed amount of time. Strictly speaking, comparing the RC of a clinical isolate to the RC of a laboratory reference strain grown in the same (drug-free) environment is a measure of *in vitro* fitness. Thus, some aspects of viral fitness can be measured *in vitro* in terms of RC [19], [20].

A low viral fitness is often held responsible for high CD4^+^ T-cell counts despite the presence of a high viral load [21]. However, although viral fitness is associated with response to antiretroviral therapy [22], it has never been applied as an additional covariate for inferring response to treatment with statistical methods. In this work, statistical models for predicting RC from genotype based on two datasets comprising genotype-RC pairs are described and validated on clinical data. The focus of this work is the assessment of the clinical relevance of predicted RC (pRC) in relation to measures of treatment response (viral load and CD4+ T-cell count).

## Materials and Methods

### Prediction of Replication Capacity from Genotype

Basis for this work were two datasets relating experimentally determined HIV RC to PR and RT sequences. In both datasets, RC was expressed as a percentage value relative to a wild-type reference strain (pNL4-3; GenBank accession no. M19921).

The first dataset, termed *Monogram*, had been the subject of study in previous works [23], [24]. The dataset comprised 317 RC-sequence pairs, and was kindly provided by one of the authors of the work by Segal et al. [24]. RC was measured using a modified version of the PhenoSense assay (Monogram Biosciences) [25]. Amino acid sequences from population-based sequencing were given for positions 4 to 99 of the PR and positions 38 to 223 of the RT. The genotypes did not contain ambiguities.

The second dataset comprised 253 RC-sequence pairs. Here, RC was measured using a novel assay developed at the University of Erlangen-Nürnberg. Genotypes were derived from patient samples by population-based sequencing and occasionally contain ambiguities. The whole PR sequence and the RT sequence up to position 220 were used. This dataset is termed *Erlangen*.

For predicting viral RC from genotype, support vector regression (SVR) models were trained on both experimental datasets. SVR was selected because it is a powerful and well-established technique that has worked well with biological sequence data in previous studies, e.g. in a work by Beerenwinkel et al. [13]. The associated computations were carried out with the R programming language and environment [26], and in particular the SVR implementation in package *e1071* [27]. All subsequent statistical analyses were also performed in R. The HIV raw genotypic information in the training datasets was encoded as numerical vectors for generating a suitable input to the SVR. Every position in an amino acid sequence was replaced by a sequence of 20 indicators. Each indicator corresponded to one of the 20 possible amino acids (in a predefined order). An indicator was set to 1 (0), if its associated amino acid was (not) present. In the case of an ambiguity at a sequence position, the indicators for the occurring amino acids are set to 1/*n*, with *n* being the number of inferred amino acids. For a protein sequence of length *m*, concatenating the indicator representations of the amino acids along the sequence led to the full indicator vector of length 20*m*. In the context of statistical modelling, every indicator constituted an input feature (predictor) for the prediction method. Constant features as well as each feature corresponding to the consensus amino acid at a certain sequence position (usually the wild-type) were removed from the indicator matrix of the training data, as such features contain no additional information. For improving robustness against outliers, the decadic logarithm of the RC percentage was used as response for the regression models rather than the RC percentage itself.

The number of predictor variables was further reduced by a feature selection step to achieve maximal performance of the regression models. Support vector regression with a linear kernel was applied for estimating feature importance. First, to obtain reasonably performing linear SVR models the cost parameter *C* was optimized by cross-validation for both datasets. Feature importance was defined as the standard score (z-score) of the coefficients in the linear SVR model. To achieve a stable feature ranking for each dataset, 100 linear SVR models from a 10×10-fold cross-validation were computed and the z-scores were averaged. The final RC prediction models were SVR models with polynomial kernels (of degree three) comprising only features with an average z-score of 1.5 and above (about 7% of the total). Leave-one-out cross-validation (LOOCV) was used for assessing the performance of RC prediction. Briefly, LOOCV uses a single observation from the dataset for validation and all remaining samples for training the model. The process is repeated for all observations from the dataset in order to obtain one prediction for every sample. In addition, a 10×10-fold cross-validation of the Monogram model was performed. Prediction error variance (the lower the better) and residual sum of squares (RSS) were computed for comparing the model performance to those previously published by Segal et al. [24] and Birkner et al. [23], respectively.

### Validation of Predicted RC on Clinical Data

The main focus of this work was the estimation of significance of viral RC in the clinical context. However, in a pre-analysis the RC prediction was validated on clinical data. More precisely, in order to validate our model beyond the correlation of measured and predicted values, pRC was challenged to confirm well-known correlations of RC with drug resistance, number of treatment lines, and treatment interruptions.

For addressing both issues, a set of clinical data in the form of *treatment change episodes* (TCEs) was extracted from the EuResist database [28]. This TCE dataset consisted of 2913 samples, each corresponding to one antiretroviral therapy. For each sample, information on the applied drug combination, the HIV genotype, and viral load (VL) before onset of the treatment (baseline) were included. For 2376 samples, baseline CD4^+^ T-cell counts were also available. Moreover, follow-up measurements of viral load and CD4^+^ T-cell count after 90 (±30), 180 (±45), 360 (±60), 720 (±90), and 1080 (±120) days were available for some samples (Table 1).

**Table 1:** Number of viral load and CD4^+^ T-cell count measurements at different points in time in the main clinical dataset.

time measure	baseline	90 days follow-up	180 days follow-up	360 days follow-up	720 days follow-up	1080 days follow-up
viral load	2913	2031	2047	1457	675	333
CD4^+^ T-cell count	2376	1621	1613	1154	526	252

As a first validation, the baseline sequences of the TCE dataset were used to study the connection between RC and drug resistance. The *cumulative resistance score* (CRS) characterizes HIV strains with respect to resistance against all 17 compounds as predicted by the geno2pheno system [13] (http://www.geno2pheno.org). Precisely, the resistance against each drug, regardless of the use in the ongoing treatment, was predicted from genotype using geno2pheno. Subsequently, the resulting fold-change values were compared to drug-specific cut-offs (already applied in [29]), and a score of 1 (“resistant” or “no activity”), 0.5 (“intermediate activity”), or 0 (“no resistance” or “full activity”) was assigned to each drug. Thus, higher scores indicated a higher overall level of drug resistance. Furthermore, the relation between resistance against single drugs and RC was studied by computing the resistance against a drug with geno2pheno and using only the sequence of the drug target for the RC prediction (the remaining sequence positions were padded according to the wild-type strain HXB2). Hence, by examining the association of resistance and predicted RC values, an indication of the validity of an RC prediction method could be gained.

In addition, 5475 genotypes from 3869 patients were extracted from the EuResist database along with the number of therapies prior to the date of genotyping. These genotypes were used to investigate the relation between pRC and cumulative treatment exposure. If multiple genotypes with the same number of previous therapies were available for the same patient, only the earliest was considered. CRS and pRC were correlated with the number of previous treatments.

A last analysis was conceived to investigate the change of pRC during treatment interruptions. The corresponding dataset comprised a total of 162 HIV genotypes from 57 patients, some of them previously included in [17]. The sequences were obtained at or around (lower quartile: 2.5 days before, upper quartile: 2 days after) the end of a treatment and at up to four different points during the therapy break. The baseline and the first follow-up genotype – usually from about two months after the end of therapy (median: 58 days, lower/upper quartile: 40/91 days) – were available for every patient, but the number of missing cases increased for each of the three later follow-ups.

### Clinical Relevance of Predicted RC

In order to study the clinical relevance of pRC, a *treatment activity score* (TAS) that summarized the activity of the ongoing antiretroviral therapy (ART) was computed. The score is closely related to the well-established genotypic and phenotypic susceptibility scores (G/PSS) [30]. Precisely, for the TAS only resistance against the compounds used in the regimen was computed and compared to the drug-specific cut-offs (as opposed to the CRS that considered all drugs regardless of their use in the regimen). Moreover, boosting of PIs by RTV was taken into account in this step, by using modified cut-offs for the PIs and disregarding the score of RTV itself. In addition, the scale of the measure was reversed compared to the CRS. Thus, the higher the score, the higher is the expected efficacy of the applied drug combination, and consequently, the lower the score, the stronger the overall resistance of the viral population against the current therapy.

The relevance of pRC for inferring a patient's response to ART was studied on the TCE dataset. In a first analysis, the TAS and pRC were computed based on the baseline genotype. The Spearman correlation between these measures and indicators of disease progression (i.e. VL and CD4^+^ T-cell count) was computed for the different time-points in the dataset. For further assessing the effects of predicted RC on prediction of therapy outcome, linear models for the prediction of changes in VL or CD4^+^ T-cell count based on the following sets of input features were trained: (i) Treatment activity expressed in terms of TAS. (ii) Resistance against the applied drugs, as predicted from genotype by geno2pheno. In place of the TAS, the raw predictions were used and the values were not combined to a single score, but used directly as inputs to the linear regression. Resistance values for compounds not included in the treatment were set to zero. Hence, the input vector comprised 17 real valued entries. (iii) The combination of applied drugs, represented as a vector of 17 binary indicators. Here no resistance was taken into account. (iv) The applied drug combination (as in iii) complemented by the TAS (as in (i)). Hence, the input vector comprised 17 binary and 1 real valued entries. In addition, each of these sets of predictors was supplemented by including both pRC features (predictions based on the Erlangen and Monogram datasets, respectively). For every time step in the TCE dataset and for each of the different feature sets, a 5×5-fold cross-validation scheme was applied to train and to evaluate linear models for predicting the change in VL and CD4^+^ T-cell count, respectively. Performance of the model was assessed by computing the correlation between the real change in VL (CD4^+^ T-cell count) and the change predicted by the model.

## Results

### Predictive Performance of RC Models

**Figure 1:**
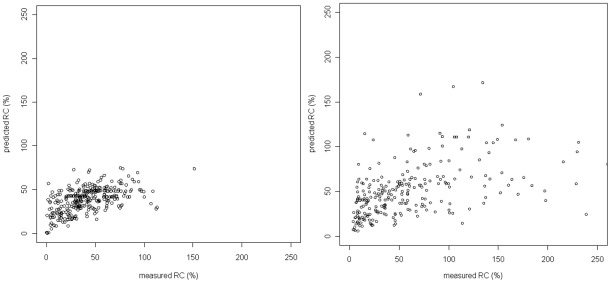
Leave-one-out cross-validation results for the Monogram data (left) and Erlangen data (right). Spearman correlations of true and predicted RC values are ρ = 0.546 (Monogram) and ρ = 0.542 (Erlangen). For the Erlangen data, seven outliers with very high measured RC would appear further to the right side of the plot, but are not shown.

Comparison of the pRC values with the true in vitro measurements resulted in Spearman correlations (*ρ*) of 0.546 for the model based on Monogram data and *ρ* = 0.542 for the model based on Erlangen data. Scatter plots of the LOOCV results are shown in Figure 1. For comparison, the geno2pheno models for the difficult to predict drugs stavudine (d4T) and zalcitabine (ddC) yielded *ρ* = 0.586 and *ρ* = 0.596, respectively. Further measures of prediction accuracy were computed for facilitating a comparison of the performance of the SVM models to previously published results. The prediction error variance (used by Segal et al. [24], the lower the better) of RC percentages (non-logarithmized) predicted by the polynomial SVR on the Monogram data is 444.1, compared to 575.6 for the best random forest model reported by Segal et al. Moreover, with a residual sum of squares (RSS) of 158.4, the cross-validated accuracy of the SVR prediction model ranks between that of the two final deletion/substitution/addition models presented by Birkner et al. (RSS of 148.3 and 178.7) [23].

### Important Mutations

Based on feature rankings derived from linear SVR models and an averaged z-score threshold of 1.5, 61 mutations were selected as significant features for each of the two RC datasets independently. Table 2 lists the top ten mutations for each dataset. The feature rankings derived from the two datasets differ widely. Only the following nine mutations present significant features for both datasets (sorted by significance): RT M184V, RT T215Y, PR L90M, PR K20I, PR I72V, RT I202V, RT V118I, PR L33F, PR L10I. For all of these mutations except for PR I72V and RT I202V, a link to drug resistance or viral fitness has been reported [20], [31]. Leaving aside the substituted amino acids and considering only mutated sequence positions, there are 22 common occurrences among the significant features of both datasets.

**Table 2:** Top ten mutations for each RC dataset according to weights derived from the initial linear SVR.

rank	Monogram mutation	influence	Erlangen rank	Erlangen mutation	influence	Monogram rank
1	RT M184V	dec.	19	RT Q207E	inc.	240
2	PR K43T	dec.	568	PR V82A	dec.	127
3	RT A158S	dec.	126	RT Y181C	inc.	150
4	PR Q92R	dec.	401	RT T215Y	dec.	18
5	PR I64L	dec.	886	RT K20I	inc.	49
6	PR K55R	dec.	602	PR I13V	dec.	132
7	PR E34K	dec.	483	RT E122K	inc.	–
8	PR I47V	dec.	366	RT L74V	inc.	141
9	PR V32I	dec.	131	RT S162C	inc.	255
10	PR P39S	dec.	141	RT T39E	dec.	267

Along with a mutation, its influence on RC compared to the wild-type is listed – “dec.” for “decreasing”, “inc.” for “increasing” – as well as its position in the feature ranking of the other dataset. With the exception of RT A158S, PR I64L, PR P39S, RT Q207E, RT E122K, RT S162C, and RT T39E, all of theses mutations are known to be associated with HIV drug resistance and/or fitness [20], [31]. In total, the two feature rankings consist of 878 mutations from the Monogram dataset and 1018 mutations from the Erlangen dataset; the difference is mainly due to the fact that fewer sequence positions are included in the Monogram genotypes. Note that the mutation RT E122K does not occur in the Monogram ranking. In the Monogram dataset, lysine (K) – not the wild-type glutamic acid (E) – is the consensus amino acid at position 122 of the RT sequence, so that E122K was removed from the training dataset in the input coding phase. The clear dominance of RC-decreasing mutations in the Monogram dataset may be partly due to the stronger bias towards low-RC samples in this dataset (median measured RC of 38.45%, compared to 46.47% in the Erlangen dataset; see also Figure 1).

### Validation of RC Prediction

**Figure 2:**
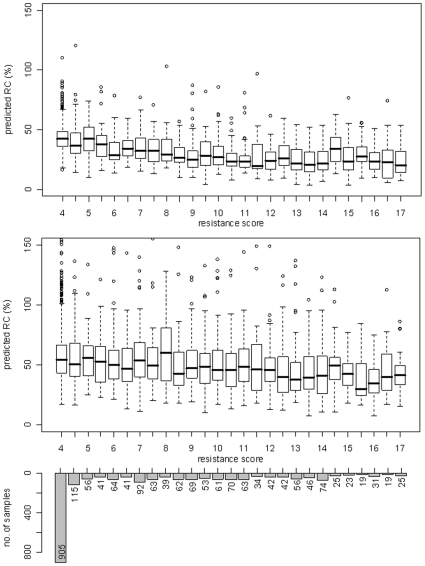
Association between predicted RC and cumulative resistance score. The underlying clinical dataset and the computation of resistance scores are described in the Methods section. For each discrete value of the score, the distribution of viral RC as predicted from the corresponding genotypes by a model based on Monogram data (top box plot) and Erlangen data (bottom box plot) is shown. The overall Spearman correlation of RC and resistance was −0.534 for Monogram and −0.233 for Erlangen RC predictions.

A graphical representation of the association of pRC and CRS for the genotypes of the main clinical dataset is depicted in Figure 2. The box plot shows the spread of the RC values for every discrete value of CRS from four to seventeen (i.e. complete resistance to all drugs). As expected, the correlation between predicted RC and overall drug resistance of a viral strain was clearly negative for both RC models. However, with *ρ = −0.534* the correlation between the Monogram model and drug resistance was more pronounced than the correlation of the Erlangen model and CRS (*ρ = −0.233*).

**Figure 3:**
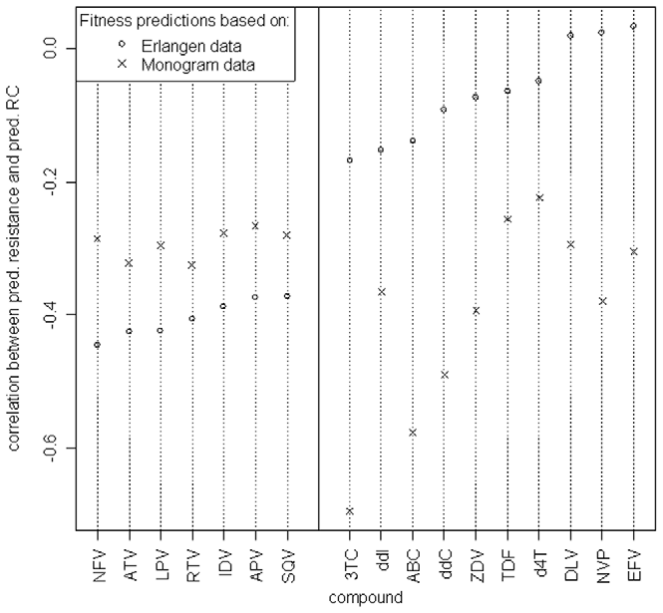
Correlation coefficients of predicted RC and predicted resistance against different antiretroviral drugs. (PIs: NFV - nelfinavir, ATV - atazanavir, LPV - lopinavir, RTV - ritonavir, IDV - indinavir, APV - amprenavir, SQV - saquinavir; NRTIs: 3TC - lamivudine, ddI - didanosine, ABC - abacavir, ddC - zalcitabine, ZDV - zidovudine, TDF - tenofovir, d4T - stavudine; NNRTIs: DLV - delavirdine, NVP - nevirapine, EFV - efavirenz). For the HIV genotypes in the main clinical dataset, drug resistances with geno2pheno, as well as RC values based on the Monogram dataset and Erlangen dataset, respectively, were predicted. In each case, two RC predictions were made: one using only the protease sequence of the sample, and one using only the reverse transcriptase sequence. (The remaining sequence positions were padded according to the wild-type strain HXB2, in order to comply with our prediction models that were derived from experimental data incorporating both PR and RT.) Spearman correlations between the protease-based RC predictions and the predicted resistances against the different protease inhibitors, as well as between RT-based RC predictions and resistance against the RT inhibitors were computed. The results are shown in the plot, with drugs ordered by increasing correlation according to RC predictions based on Erlangen data.

Figure 3 depicts the correlation of predicted RC and predicted resistance against individual antiretroviral drugs. For almost all compounds negative correlation coefficients, independent of the dataset used to derive the RC prediction model, were observed. Exceptions were the NNRTIs delavirdine (DLV), nevirapine (NFV), and efavirenz (EFV), which exhibited correlations slightly above zero if RC was predicted with the Erlangen model. Besides, there existed a difference with respect to the RC prediction model and the drug classes. For protease inhibitors, correlation coefficients of resistance and RC were consistently lower for the Erlangen model than for the Monogram model, and the inverse was true for reverse transcriptase inhibitors.

**Figure 4:**
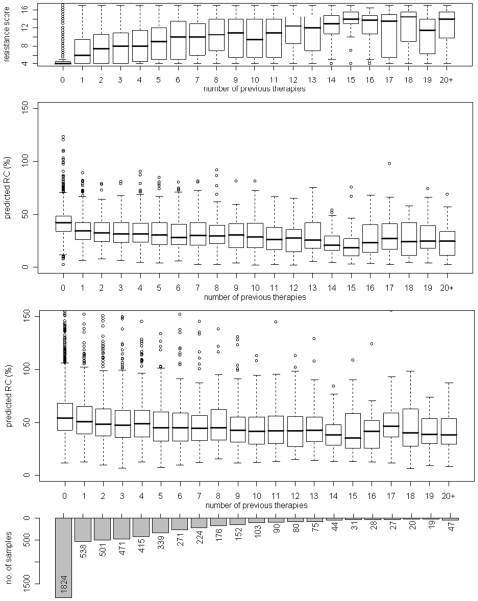
Relationship between drug resistance, predicted RC and treatment experience. Based on 5475 genotypes from the EuResist database, the distribution of resistance scores and predicted viral RC for different numbers of previous therapies (completed before the date of genotyping) is shown. In the middle box plot, RC was predicted from a model trained on Monogram data; below, RC predictions are based on Erlangen data. The overall Spearman correlation between the number of previous therapies and the resistance score is 0.560; for the number of therapies and predicted RC, we obtain correlation coefficients of −0.336 (Monogram predictions) and −0.231 (Erlangen predictions).

Figure 4 displays the distribution of pRC and CRS for different numbers of previous treatments. Patients with 20 or more treatments formed a single group. The largest group of patients were the therapy-naïve patients. CRS showed a strong positive correlation with the number of previous therapies (*ρ* = 0.560) and, as expected, the pRC was negatively correlated with the extent of treatment exposure (*ρ* = −0.336 for the Monogram model and *ρ* = −0.231 for the Erlangen model). Although the correlations were not as pronounced as the one observed between CRS and treatment experience, the distribution of pRC in the naïve patients and the patients with e.g. 9 previous treatments was significantly different according to a t-test (p-value of <2.2 • 10^−16^ and 1.699 • 10^−9^ for the Monogram and Erlangen model, respectively).

**Figure 5:**
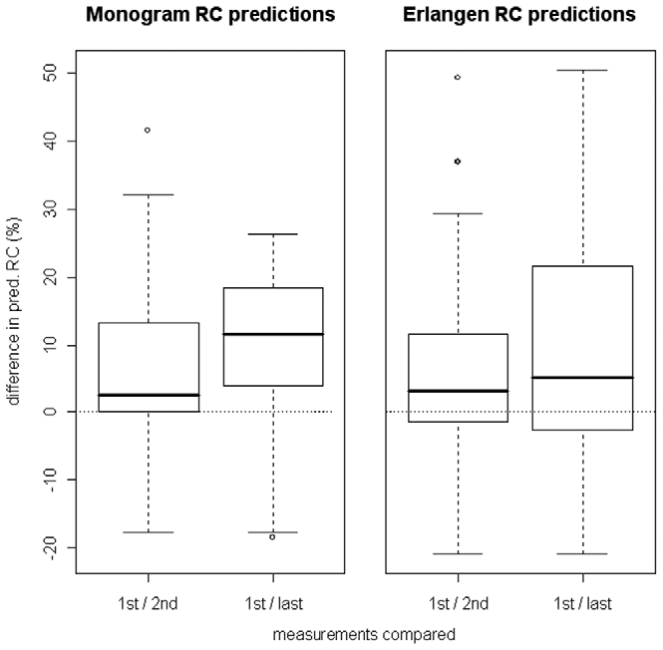
Changes in viral fitness during treatment interruption. Models derived from the Monogram dataset (left side of the plot) and Erlangen dataset (right side) were used to predict the viral RC corresponding to the genotypes in the treatment interruptions dataset described in Materials and Methods. Shown in the plot are the differences between baseline RC and the first follow-up RC (n = 56) - corresponding to a genotype from about two months into the therapy break -, as well as the differences between baseline RC and the last follow-up RC (n = 30; only if there was more than one follow-up measurement; typically from five to six months into the break).

For HIV genotypes obtained during treatment interruptions, the models clearly tended to predict RC values that increased over time (i.e. difference between first and second measure compared to difference between first and last measure increased), as shown in Figure 5. According to a paired t-test, the distribution of the baseline measurement and first (*n* = 56) [last (*n* = 30)] measurement during the therapy break were significantly different for the Monogram and Erlangen model with p-values of 0.02919 [0.01402] and 0.00962 [0.02000], receptively.

### Clinical Relevance of Predicted RC

**Figure 6:**
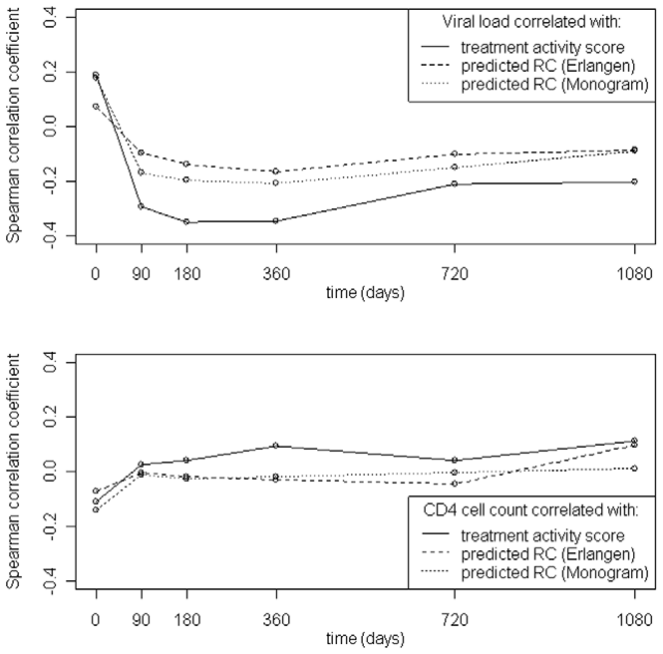
Spearman correlations between clinical markers (top: viral load, bottom: CD4+ T-cell count) and potential predictors of clinical response (predicted RC and TAS) at different points in time during therapy. Correlation coefficients were computed from the TCE dataset.

**Figure 7:**
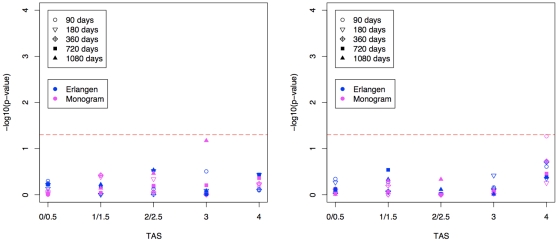
The figure depicts the p-values obtained from a one-sided t-test testing whether predicted RC is lower in patients succeeding a treatment than in patients failing a treatment. Patients are grouped according to the predicted activity (TAS) of the regimen. Virologic success (left) was defined as reaching the limit of detection, i.e. 50 copies of HIV RNA per one ml blood. Immunologic success (right) was defined as an increase of CD4+ T-cells per one µl blood by 50% or more. Different symbols and colors are used to indicate different time-points of the follow-up measurement and different RC prediction models, respectively. The red horizontal dashed bar represents the 5% significance threshold.

Figure 6 summarizes the correlation between pRC or TAS and VL (CD4^+^ T-cell count) at different time points. In general, correlation coefficients were low. A positive (negative) correlation between pRC and VL (CD4^+^ T-cell count) was observed only for the baseline measurements. At 90 days after therapy start, the direction of the correlation between pRC and VL was inversed. The strongest negative correlation was observed at about one year of treatment. From then on the strength of the correlation steadily decreased. However, at all time points the VL showed equal or stronger correlation to TAS than to pRC. For 90 days and later correlation of pRC with CD4^+^ T-cell count was close to zero and correlation of TAS and CD4^+^ T-cell count was slightly positive. Figure 7 summarizes the results of a one-sided t-test testing whether pRC was lower in patients succeeding a treatment than in patients failing a treatment. Patients were grouped with respect to the activity of the treatment and the time of the follow-up measurement. Treatment success was defined by a decline of VL below the limit of detection (i.e. 50 copies per ml) or increase of CD4^+^ T-cells by 50% or more compared to the baseline measurement. No group exceeded the 5% significance threshold.

**Figure 8:**
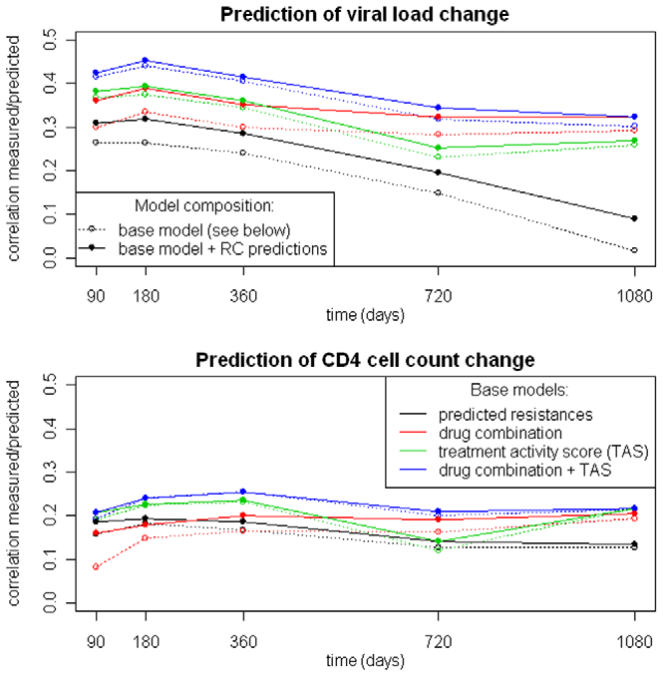
Performance of linear models for the prediction of changes in viral load (top) and CD4+ T-cell count (bottom). Based on the main clinical dataset, linear models were trained using different sets of input features: 1. predicted resistances against applied drugs, 2. applied drug combination, 3. TAS, 4. drug combination and TAS. Also, for each of these variants, a model that includes two predicted RC values (based on the Monogram and Erlangen datasets) as additional features was considered. This setup allowed us to compare different properties with respect to predictivity of clinical response and to assess the significance of RC in this context.

The upper panel of Figure 8 shows the results of predicting the change in VL between baseline and different time points during treatment. The models using pRC values as additional features performed better in general. However, the magnitude of improvement was dependent on the base model. The most pronounced improvements were observed for the base models using predicted (raw) resistances and binary indicators for the drug combination, respectively. For models that included TAS as a predictor, improvements were only moderate. The model that used binary drug indicators and pRC as predictors performed as well as the one using TAS and pRC in the first year of treatment, and afterwards even outperformed the TAS and pRC-based model. In general, the best performance was achieved when predicting the change in VL at 180 days, afterwards predictive performance decreased for all models. Including pRC as an additional feature improved the models for predicting VL change at 180 days based on raw fold-resistance values, indicators for the drug combination, or the TAS significantly (t-test; p-values ranging from 0.0247 to 4 • 10^−6^).

Prediction of change in CD4^+^ T-cell count achieved worse performance than prediction of change in VL (lower panel in Figure 8). The peak of predictive performance was reached at one year of treatment. For later time points the predictive performance ceased. Again, models using pRC as an additional predictor achieved comparable or better performance at all time points. As in the case of VL change prediction, the major improvement was observed for the model that used only binary drug indicators. However, none of the improvements of the 360 days models reached statistical significance.

## Discussion

Based on experimental data, viral RC could be predicted from HIV genotypes with moderate accuracy. The prediction accuracy of the SVM models compare well to previously published results. Moreover, results on the clinical data showed that this accuracy was sufficient to confirm some pre-existing notions about RC derived from actual data. Thus, in general it is possible to draw meaningful conclusions about the clinical significance of RC based on RC predictions made by the models discussed here. The prediction step added a source of uncertainty to the approach, which was certainly a drawback. However, it also allowed for leveraging the wealth of treatment data for which genotypes, but no RC measurements, are available.

The validation on clinical data showed an inverse relationship of pRC to drug resistance against single drugs and to the CRS. Since most mutations that confer resistance are linked to an impairment of viral RC, this type of association was expected. The clear separation of drug classes in Figure 3 for the Erlangen model could be related to increased number of viruses with highly mutated PR in the Erlangen data compared to the Monogram data, e.g. using the mutation list from [31] 61% (25%) of the sequences in the Erlangen dataset had at least one (five) protease mutations compared to 28% (3%) in the Monogram dataset. Correlation between overall treatment experience and RC was clearly negative, i.e. RC decreased when the number of previous treatments increased. In general, a change of therapy accompanied by a viral genotype marks a failure of the previous treatment, which in turn was caused by resistance against the applied drugs. These results confirmed the assumption that drug resistance has a negative impact on RC. Moreover, the observed increase of predicted RC during treatment interruptions complied with the established understanding of the implications of “drug holidays”. Without the selective pressure exerted by antiretroviral medication, the HIV population will be overgrown by strains that are less resistant but exhibit higher RC, because those strains have a fitness advantage in the drug-free environment. Evolution during the treatment interruption of a subset of viruses was previously analyzed in detail, and re-emergence of wild-type variants (defined as loss of all drug resistance mutations) was observed only in about 18% of the cases [17]. Hence, re-emergence of complete wild-type variants is an unlikely explanation for the observed increase in pRC. Altogether, these observations support the validity of our RC prediction method.

When studying the relationship between pRC and markers of treatment response, only the measurements at baseline confirmed assumptions about the direction of the relation. Later measurements showed a relation that was inverse to the initially expected one. However, this makes sense considering that, as an antiretroviral therapy begins to take effect, the activity of the treatment becomes the main influence on the replication of the virus population. In other words, under the impact of the medication, viral fitness is determined mainly by resistance against the applied drugs, rather than by the replication capacity. Since drug resistance mutations were usually associated with a negative influence on RC, as previous results showed, the initial correlations between RC and the clinical markers could not be observed at the follow-up points. Moreover, in patient groups receiving equally active regimens, pRC was not lower in patients with virologic or immunologic success than in patients experiencing a treatment failure across all follow-up times and activity classes. In addition, this result contradicts the general belief that low viral fitness may occasionally help to maintain high levels of CD4^+^ T-cells in the absence of an effective treatment.

The decrease of performance for models that predict change in VL (CD4^+^ T-cell count) for time points after 180 (360) days might be explained by the emergence of new mutations during the course of therapy. Hence, the baseline HIV genotype (from which most predictors are derived) ceased to determine the clinical response at later points in time. This notion seems to be substantiated by the fact that the decrease in predictive power was less (in the case of VL) or none (in the case of CD4^+^ T-cell count) for predictions based on the combination of applied drugs, which were independent of the genotype. Moreover, it was surprising that predicted resistances against the applied drugs were less indicative of clinical outcomes than TAS, which was in fact a summary of the resistances. However, the drug-specific cut-offs that were applied for computing TAS were based on clinical knowledge not present in the raw resistance values. Remarkably, the model based on TAS (TAS and pRC) performed worse than (similar to) the model based simply on drug indicators and pRC. Here pRC seems to summarize the resistance information without the need of providing clinically optimized cut-offs. Perhaps most important for the core question of this work is the fact that the inclusion of pRC features could indeed improve the prediction of clinical response. The amount of improvement differed between model formulations, but was quite consistent for different time steps. However, for linear models that incorporated TAS as a predictor the performance gain conferred by additional RC features was negligible.

### Conclusion

Previous results suggested that RC is significantly associated with treatment outcome under certain conditions, even though it is not predictive on its own [32]. The findings in this study support these conclusions to some extent, but also clearly show that pRC was rather uninformative if available information on drug resistance and medication was fully utilized. Examples are the linear models for predicting change in VL after 180 days of treatment. The two pRC features in the model were statistically highly significant (t-test yielding p-values of 1.81 • 10^−5^ and 0.0011 for Monogram and Erlangen predictions, respectively), but the resulting improvement in predictive performance, compared to the model without pRC, was marginal. More generally, the significance level of a predictor in the models compared did not necessarily attest to the amount of gain in prediction quality that might arise through the inclusion of this predictor.

There is clearly some room for improvement of RC prediction models, and the lack of large high-quality genotype-RC datasets for training purposes was certainly a limiting factor of this study. Existing prediction methods, like the one proposed here, could benefit from datasets as large as those currently used for the related problem of predicting HIV drug resistance from genotype, where 1,000 samples per drug are not uncommon [13], [14]. However, it remains doubtful if increased quality of RC predictions would have led to different conclusions from these analyses about the clinical significance of RC. After all, robust associations of pRC with drug resistance, overall treatment experience, and treatment interruptions were observed. Since RC and drug resistance are highly correlated, and since the minor effect of RC on clinical response pales in comparison to the dominant role of resistance, there seems to be little benefit in considering RC as an additional therapeutic indicator. Thus, the use of RC for treatment selection in clinical practice may play only a minor role.
